# Augmented Intrarenal and Urinary Angiotensinogen in Diabetic Nephropathy: The Role of Isoflavones

**DOI:** 10.3390/ijms26041443

**Published:** 2025-02-08

**Authors:** Masumi Kamiyama, Kotoe Iijima, Rema Okuzawa, Ruka Kawata, Airi Kimura, Yuki Shinohara, Ayana Shimada, Mika Yamanaka, Ayuka Youda, Tamami Iwamoto

**Affiliations:** Department of Food and Nutrition, Jumonji University, 2-1-28, Sugasawa, Niiza 352-8510, Saitama, Japan

**Keywords:** angiotensinogen, diabetic nephropathy, isoflavones, polyphenols, renin–angiotensin system

## Abstract

The circulating renin–angiotensin system (RAS) is an endocrine system with key functions in maintaining blood pressure, fluid volume, and electrolytes. The RAS in the kidney (intrarenal RAS) plays a critical role in the onset and progression of kidney diseases. However, the mechanism underlying the onset and progression of diabetic nephropathy in relation to the expression and secretion of angiotensinogen (AGT) in the kidneys remains unclear. In this review, we present an overview of the intrarenal RAS and its role in diabetic nephropathy, as well as reviewing the evidence for the use of urinary AGT as a biomarker of this system in diabetic nephropathy. We also describe the roles of isoflavones in the context of diabetic nephropathy. The considered studies show that the intrarenal RAS—especially AGT—plays a diversified role in diabetic nephropathy; for instance, the increase in AGT due to oxidative stress is suppressed by polyphenols with antioxidant capacity, which is thought to affect the progression of diabetic nephropathy. Therefore, clarification of how polyphenols affect the onset and progression of diabetic nephropathy may provide insights into new treatments for this illness.

## 1. Introduction

Diabetic nephropathy is a major healthcare challenge. It occurs in up to 50% of diabetic patients, is the leading cause of end-stage kidney disease, which requires treatment by dialysis or kidney transplantation, and is associated with significantly increased cardiovascular morbidity and mortality. Moreover, it negatively impacts the quality of life and social environment of patients, as well as putting a strain on national health budgets [1]. Diabetic nephropathy develops as a result of the interplay of hemodynamic and metabolic factors [1]. The hemodynamic factors that contribute to the development of diabetic nephropathy include elevated systemic and intraglomerular pressures and the activation of vasoactive hormonal pathways, including the renin–angiotensin system (RAS) [2] and endothelin [3]. These hemodynamic pathways activate protein kinase C (PKC) [4], mitogen-activated protein (MAP) kinases [5,6], nuclear transcription factors (e.g., NF-κB), and various growth factors, such as pro-sclerotic cytokines, transforming growth factor (TGF)-β, permeability-enhancing growth factor, and vascular endothelial growth factor (VEGF) [7]. Glucose-dependent pathways are most often triggered in diabetic kidneys, leading to increased oxidative stress, renal polyol formation [8], and the accumulation of advanced glycation end products [9]. The combination of these pathways ultimately leads to increased renal albumin permeability and extracellular matrix accumulation, resulting in increased proteinuria, glomerulosclerosis, and, ultimately, tubulointerstitial fibrosis [10].

The RAS was discovered as a humoral factor related to systemic blood pressure and body electrolytes. Studies have shown that the RAS in the kidney plays an important role in the onset and progression of kidney disease [11,12,13]. Angiotensinogen (AGT) is a precursor of angiotensin II and is the most upstream factor of the RAS. Research has demonstrated that the amount of AGT excreted in urine is useful as an early biomarker of diabetic nephropathy [14,15,16]. AGT has been linked to an increase in reactive oxygen species in diabetic nephropathy. Polyphenols with antioxidant properties are thought to affect the progression of diabetic nephropathy through the elimination of reactive oxygen species; however, at present, the details of the underlying mechanism are unclear.

The purpose of this review is to show that urinary angiotensinogen is a marker of early diabetic nephropathy, that oxidative stress increases the expression and secretion of angiotensinogen in the kidney, and that polyphenols with antioxidant effects affect AGT expression in the kidney and to clarify the possibility that soy isoflavones—which have been shown to have strong antioxidant effects—may suppress the progression of diabetic nephropathy through their effects on the expression and secretion of AGT.

## 2. The RAS

**Figure 1:**
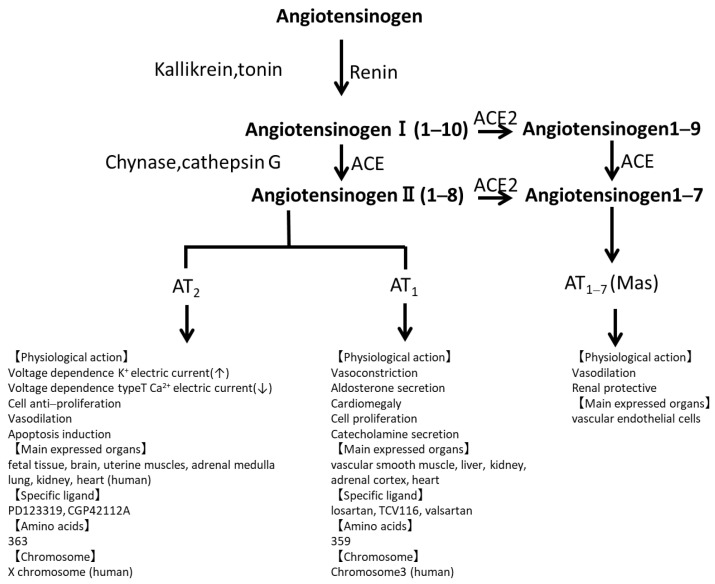
The RAS cascade.

The RAS mechanism is depicted in Figure 1 [17]. The renin enzyme is secreted from juxtaglomerular cells in the kidneys into the blood. Renin cleaves its target protein AGT, which is produced in the liver and is continuously present in plasma, into the inactive peptide angiotensin I. The balance between vasoconstrictor and vasodilator effects is determined by the actions of angiotensin II and angiotensin I–VII. The production of angiotensin II is dependent on the availability of AGT and angiotensin I, as well as the activities of renin, the angiotensin-converting enzyme (ACE), and ACE-independent enzymatic pathways including chymase and cathepsin G (a lysosomal enzyme with protease activity). Angiotensin I–VII are formed either directly from angiotensin II being hydrolyzed by ACE2 or indirectly from angiotensin I, with an intermediate step involving the formation of angiotensin I–IX being hydrolyzed by ACE2 and ACE in sequence. The function of angiotensin II is determined by signaling via angiotensin II type 1 and type 2 receptors [18] and the putative angiotensin I–VII receptor Mas [19,20,21,22,23].

## 3. The Intrarenal RAS

In addition to the circulating RAS, research has demonstrated the presence of local RASs [24] in numerous tissues and systems, including skeletal muscle [25], bone [26], the kidneys [18,27,28], the cardiovascular system [29], the brain [30], and intervertebral disk tissues [31]. The intrarenal RAS is notable, as all components required for the generation of angiotensin II, angiotensinogen, renin, angiotensin I, and ACE are present in the kidney. The renal RAS has important functions, including the regulation of blood pressure, renal cell growth, and the production of glomerulosclerosis, which is involved in the development of renal fibrosis. Research in experimental animal models and transgenic mice has demonstrated the involvement of AGT in the activation of the RAS [32,33,34,35,36].

## 4. Intrarenal Localization of AGT, Renin, and ACE

AGT is localized primarily at the mRNA level [37], while immunoreactive AGT [27] has been identified in the proximal tubules. Strong *AGT* mRNA expression was detected in the proximal straight tubules. The proximal convoluted tubules and proximal straight tubules exhibited positive immunostaining for AGT. A weak expression of AGT protein was also observed in glomeruli and vasa recta, while no AGT expression was observed in distal tubules and collecting ducts. This evidence suggests that AGT is constitutively secreted in the proximal straight tubules in the same manner as it is secreted in the liver [38].

*Renin* mRNA has been detected in cultured proximal tubular cells, and a low concentration of renin was found in proximal tubule fluid [39] in rats [40,41,42,43]. The expression of *ACE* mRNA and proteins was identified on brush border membranes of proximal tubules of kidneys. ACE is present in proximal and distal tubular fluid. The ACE2 protein is expressed in proximal tubule cells, glomerular podocytes, and tunica media of renal arterioles [44].

## 5. Urinary AGT as a Biomarker of Intrarenal RAS in Diabetic Nephropathy

Microalbuminuria is the most commonly used clinical indicator of diabetic nephropathy in both type 1 and type 2 diabetic patients [45,46]. Diabetic nephropathy was thought to be a unidirectional process, which starts with microalbuminuria and leads to end-stage renal failure [47]. Therefore, the presence of albumin in the urine has been considered predictive of the subsequent development and clinical progression of diabetic nephropathy. However, it has recently been shown that a large proportion of type 1 diabetic patients with diabetic nephropathy reverted to normoalbuminuria, of which one-third of them exhibited reduced renal function even during the macroalbuminuric stage [48]. Therefore, a more sensitive and specific marker for diabetic nephropathy, rather than urinary albumin excretion, is needed.

Estimated factors for diabetic nephropathy are shown in Table 1. Various proteins, such as urinary type IV collagen, adiponectin, and advanced glycation end products, have been suggested as potential markers of diabetic nephropathy. As glomerular injury markers, oxidized albumin, type IV collagen, and ceruloplasmin have been reported. Serum oxidized albumin levels may be useful for the early diagnosis of diabetic kidney disease and predicting renal outcomes. On the other hand, α1- microglobulin (MG), kidney injury molecule (KIM)-1, L-fatty acid binding protein (FABP), neutrophil gelatinase-associated lipocalin (NGAL), and N-acetyl-beta-D-glucosaminidase (NAG) have been reported as markers of renal tubular injury. NGAL, as a biomarker of renal tubular injury, is upregulated in the distal tubules and collecting ducts and has been extensively evaluated for its involvement in acute kidney injury. It is a 25 kDa glycoprotein containing 178 amino acids and belongs to the lipocalin superfamily, it is a component of certain granules, and it is present in neutrophils as part of the NGAL–gelatinase complex. It is involved in antibacterial defense mechanisms and is upregulated in systemic bacterial infections, and it plays a protective role in epithelial injury due to its antiapoptotic effect. It is not produced by burned-out nephrons and is, therefore, considered to be a marker of active injury and represents the salvageable nephron mass. Its utility as a biomarker of chronic kidney disease and subsequent diabetic nephropathy has been suggested. Other tubular injury markers have recently been discovered; for example, increased levels of KIM-1, NGAL, NAG, and cystatin C are believed to indicate proximal tubular injury, while cardiac heart-type fatty acid-binding protein (H-FABP) is thought to indicate distal tubular injury. These tubular injury markers have been extensively studied in terms of predicting the development of acute kidney injury after various nephrotoxic insults, such as ischemia, sepsis, and the administration of contrast agents during cardiac surgery. Few studies have been performed in patients with chronic kidney disease. NAG is a marker of proximal tubular injury in diabetic patients and non-diabetic control subjects, and this marker is related to the severity of kidney disease and the estimated glomerular filtration rate, as assessed with respect to albuminuria. Inflammatory cytokines, such as interleukin (IL)-6, IL-8, IL-10, and IL-18, have also been reported in this context; for example, multiple studies have demonstrated that IL-6 signaling contributes to the progression of diabetic nephropathy. It has been observed that type 2 diabetes mellitus patients with diabetic nephropathy have higher levels of IL-6 in the bloodstream than their counterparts without diabetic nephropathy, suggesting a significant association between IL-6 and the development and progression of diabetic nephropathy. IL-6 has become an attractive focus in diabetes research due to its multiple functions in regulating glucose balance. IL-6 promotes insulin effectiveness through enhancing glucose clearance in the liver and skeletal muscle during exercise. Growth factors, such as TGF-β and connective tissue growth factor (CTGF), are biomarkers reflecting both glomerular and tubulointerstitial hallmarks of diabetic kidney disease. TGF-β is a member of the TGF-β superfamily, which encompasses a number of structurally related proteins that can be classified into several subfamilies, including TGF-β, activins/inhibins, and bone morphogenetic proteins. TGF-β ligands are widely expressed in a variety of cell and tissue types, but they exist in three isoforms: TGF-β1, 2, and 3. TGF-β ligands are synthesized as larger precursor proteins, the N-terminal portion of which is cleaved to release the mature C-terminal ligand in the form of a homodimer. The cleaved N-terminal peptide binds to the C-terminal ligand. The activity of the mature TGF-β homodimer is sequestered by a latent TGF-β binding protein, termed latent TGF-β. Active TGF-β can be released through enzymatic digestion or an acidic microenvironment. Leukocyte adhesion to endothelial cells is a crucial step in the development of vascular complications. Molecules involved in adhesion are involved in inflammation, endothelial dysfunction, and the development of microvascular (neuropathy, retinopathy, and nephropathy) and macrovascular complications through a series of steps controlled by adhesion molecules on leukocytes and endothelial cells. vascular cell adhesion molecule (VCAM)-1, intercellular adhesion molecule (ICAM)-1, and selectins (E-selectin, L-selectin, and P-selectin) are the main cell adhesion molecules involved in the development of microvascular complications. In type 2 diabetes patients with microvascular disorders, the expression of cell adhesion molecules cannot be controlled, so the development of microvascular complications may be prevented by examining the changes in the expression levels of cell adhesion molecules. In patients with chronic kidney disease, renal dysfunction increases mortality, especially when the glomerular filtration rate (GFR) is reduced. Cardiovascular risk factors such as hypertension and diabetes are common, and cardiovascular complications associated with uremia-related risk factors such as inflammation, endothelial dysfunction, oxidative stress, fluid overload, and vascular calcification adversely affect disease control, especially in end-stage renal disease. Advanced glycation end products (AGEs), such as carboxymethyllysine (CML), carboxyethyllysine (CEL), pentosidine, pyrraline, imidazolone, and crosslin, are generated by the covalent binding of amino groups to sugars or sugar derivatives during the nonenzymatic Maillard reaction. In patients with chronic kidney disease, the formation of AGEs is promoted by hyperglycemia, oxidative stress, and inflammation, and chronic kidney disease worsens as the kidney’s ability to eliminate AGEs decreases. High levels of pentosidine in the blood have been reported to be associated with inflammation, malnutrition, cardiovascular disease, and poor clinical outcomes. However, the contribution of pentosidine to the development of cardiovascular events and mortality in patients with chronic kidney disease has been questioned [14], and traditional risk factors in patients with end-stage renal disease have been reported to be more important for cardiovascular outcomes than elevated AGEs levels [14,15,16,49,50,51,52,53,54,55,56,57,58,59,60,61,62,63,64,65,66,67,68,69,70,71,72,73,74,75,76,77,78,79,80,81,82,83,84,85,86]. However, such divergent findings have complicated the search for a reliable biomarker. A clinical trial has demonstrated that the activation of the intrarenal RAS had a potential role in the mechanism of diabetic nephropathy. Angiotensin-converting enzyme inhibitors, angiotensin II receptor blockers, or a dual blockade of the RAS were shown to provide renoprotection in patients with type 1 or type 2 diabetes, and some reports have shown that such results are independent of systemic blood pressure changes.

We previously examined whether increased urinary AGT excretion is present prior to the onset of urinary albumin in streptozotocin-induced type 1 diabetic mice; we found that urinary AGT may be useful as an early biomarker of the activation of the RAS in experimental type 1 diabetes. Urinary AGT excretion is higher in patients with type 1 diabetes compared with control subjects [14]. Despite the importance of the RAS in the development of diabetic nephropathy, the significance of the intrarenal RAS—especially the role of AGT—in the early stage of type 1 diabetic nephropathy has not yet been fully revealed. We investigated urinary AGT levels as a candidate marker of the activation of the RAS in type 1 diabetes. Streptozotocin-induced type 1 diabetic mice showed typical symptoms of diabetes mellitus, while insulin treatment ameliorated these changes, in agreement with short-term investigation studies. We observed increases in the excretion levels of urinary AGT and albumin in diabetic mice compared with the control and insulin-treated groups. These observations indicate that the urinary excretions of AGT and albumin were increased in the streptozotocin-treated mice compared with the control animals. Notably, the augmented excretion levels of AGT in urine were apparent before the development of increased albumin levels. This could be due to the fact that urinary AGT levels are highly sensitive to the onset of nephropathy. Furthermore, our data showed that the ratio of urinary AGT to urinary albumin was significantly higher in the streptozotocin group than in the control group. As insulin treatment mitigated the increased excretion of urinary albumin and urinary AGT, we speculate that the increased glucose level induced elevated urinary albumin and AGT excretions. Our result suggesting that AGT may be a useful biomarker has been confirmed by other researchers.

The upregulation of AGT levels may lead to elevated angiotensin peptide levels. Our studies in diabetic models have documented the involvement of AGT in the activation of the RAS [87]. While intrarenal renin expression is also increased in diabetic animals, some in vitro reports using a rat immortalized renal proximal tubular cell line have shown that high glucose levels augmented AGT gene expression [16,88]. Under normal conditions, there is no excretion of glucose in the urine. This is because almost 90% of filtered glucose is reabsorbed by sodium-glucose cotransporter-2 (SGLT2) in the proximal tubule, and the remaining 10% is reabsorbed by SGLT1 in the descending proximal tubule. In type 2 diabetes, it is known that the expression of SGLT2 increases, increasing glucose reabsorption. In addition, as diabetes worsens, renal function declines. Diabetic nephropathy is the main cause of chronic kidney disease and end-stage renal disease, resulting in a condition requiring dialysis. Diabetic patients have high urinary albumin excretion, which is due to a reduced glomerular filtration rate due to a high glucose reabsorption capacity in the proximal tubule. Therefore, prevention of chronic kidney disease leads to a reduction in glucose reabsorption in the kidneys of diabetic patients.

## 6. The Role of Isoflavones in the Development of Diabetic Nephropathy

The oxidative modification of lipids, proteins, and nucleic acids by reactive oxygen species plays a pivotal role in a wide range of common diseases and age-related degenerative conditions. Increases in antioxidative capacity are believed to play a protective role against such oxidative damage. Many studies have shown that oxidative stress, fibrosis, and inflammation may play key roles in the progression of diabetic nephropathy [89]. Oxidative stress occurs in the early stage of diabetic nephropathy and triggers diverse pathological pathways in almost all renal cells. Fibrosis is a prominent and fundamental feature of diabetic nephropathy and inflammation and appears to play a major role in the onset and development of renal fibrosis. We focused on the role of angiotensinogen and oxidative stress in diabetic nephropathy. Xiao et al. recently showed that the in vitro and in vivo treatment of diabetic models with epiberberine (an alkaloid isolated from *Coptidis rhizoma*) resulted in an improvement in diabetic nephropathy due to its effects in reducing angiotensinogen, TGFβ1, and SMAD family member (Smad)2 expression [90].

The RAS was discovered as a humoral factor that is related to systemic blood pressure and body electrolytes. The intrarenal RAS in the kidney plays important roles in the onset and progression of kidney disease. AGT is a precursor of angiotensin II, which is located at the most upstream part of the RAS. However, the details regarding how AGT expression and secretion in the kidney are controlled in the progression of diabetic nephropathy remain unclear.

Polyphenols are secondary metabolites derived from plants [91]. Polyphenols can be divided into flavonoids, phenolic acids, lignans, tannins, and stilbenes according to their structural differences. Isoflavones are a type of polyphenol found in legumes, including soybeans, chickpeas, fava beans, pistachios, peanuts, and other fruits and nuts. Polyphenols with antioxidant properties are thought to affect the progression of diabetic nephropathy through the elimination of reactive oxygen species, but the detailed mechanism remains unclear. We previously investigated the effects of polyphenols, which have high antioxidant properties, on AGT—an early marker of diabetic nephropathy [92]. No studies had examined whether there are any food ingredients that suppress AGT expression in the kidneys and, thus, these findings have important implications for the development of potential dietary therapies for diabetes. The “question” of this study is whether soy isoflavones—a type of polyphenol—are involved in the mechanism of the progression of diabetic nephropathy through the expression and increased secretion of AGT as an early marker of diabetic nephropathy. While some reports have shown that sardine and wakame seaweed peptides inhibit ACE, indicating that these food components affect the RAS, whether there are food components that suppress the expression of AGT is unclear.

Our studies demonstrated that the hydrogen-peroxide-based stimulation of renal mesangial cells increases AGT expression and secretion, indicating AGT as an important molecule in kidney disease. Recent reports have demonstrated that the AGT-mediated TGF-β/Smad2 expression mechanism is involved in renal fibrosis [90]; this study also examines this mechanism. Considering these reports, this study clarifies the significance of food components regarding the expression of AGT in the RAS in the kidney. To explore new possibilities for such foods or food components, we examined how AGT is involved in the progression of diabetic nephropathy and how soy isoflavones act to suppress this fluctuation.

Isoflavones exert antioxidant functions through their free radical scavenging ability, their ability to reduce low-density lipoproteins and the susceptibility of DNA to oxidative stress, and their ability to boost the activity and expression of antioxidant enzymes. Isoflavones have been linked to decreased risks of cardiovascular disease, osteoporosis, endocrine-responsive cancer, and menopausal symptoms, partly because of their possible antioxidant activities.

Isoflavones, together with coumestans and prenylflavonoids, belong to the group of flavonoid phytoestrogens naturally found in non-steroidal phenolic plant compounds. Isoflavones in the legume family include inactive hydrophilic glycosides (e.g., daidzein, genistin, and glycin in soybean) and 4′-methylated lipophilic derivatives (e.g., formononetin and biochanin A in red clover). The half-life of isoflavones is about 9 h for daidzein and about 7 h for genistein. Some aglycones are further structurally altered by the intestinal flora; for example, genistein is converted to dihydrogenistein, which is converted to p-ethylphenol and 6-hydroxy-O-desmethylangolensin. Daidzein is metabolized to dihydrodaidzein, which is converted to equol. Equol has estrogenic effects and strong antioxidant properties. When soy products are eaten, the soy isoflavones in soybeans reach the intestine, where they are metabolized by intestinal bacteria to produce equol. Therefore, quantifying the amount of equol excreted in urine can tell us how much protein was consumed from soy products. Certain intestinal bacteria classified into the genera Adlercreutzia, Eggerthella, and Slackia are known to be equol-producing bacteria that can carry out the entire series of metabolism from daidzein to equol. The production of equol depends on the composition of the intestinal flora and is also said to be due to genetic predisposition, but approximately half of adults do not excrete equol in the urine. Some reports have shown the protective effects of isoflavones against diabetes-induced renal damage (Table 2) [93,94,95,96,97,98,99]. While there have not been many reports in this line, some have shown that these compounds can inhibit fibrosis and inflammation; however, the underlying mechanism remains unclear. There are also reports that polyphenols such as catechin and quercetin suppress the progression of diabetic nephropathy—this is an area of research that is expected to see further development in the future [100,101,102,103,104].

A comprehensive review of existing meta-analyses of randomized controlled trials focusing on the effects of dietary interventions on the incidence of diabetic nephropathy suggested that probiotics, vitamin D, soy isoflavones, coenzyme Q10, dietary polyphenols, antioxidant vitamins, or salt-restricted diets could significantly improve diabetic nephropathy. A comprehensive review revealed that soy isoflavone supplementation significantly improved blood urea nitrogen (BUN), fibrinogen (FBG), total cholesterol, total glucose, low-density lipoprotein cholesterol (LDL-C), and 24 h urinary protein compared with no soy isoflavone supplementation. However, soy isoflavone supplementation in diabetic patients did not significantly improve body weight, serum creatinine (Scr), creatinine clearance (CrCl), glomerular filtration rate (GFR), and high-density lipoprotein cholesterol (HDL-C). Phytosterols contained in soybeans can competitively inhibit cholesterol synthesis in the body and lower serum cholesterol levels. To improve renal function, soy foods can lower 24 h urinary protein levels. Replacing part of the animal protein in the diet with soy protein can improve the hemodynamic function of the kidney and reduce urinary protein excretion. Soy protein itself is a high-quality protein, and the price of raw materials is relatively high. After the digestibility of soy foods has been greatly improved, soy protein and animal protein play the same nutritional role. In addition, soy protein is lower in fat than animal protein, which can help diabetic patients control the total calorie intake in the diet and reduce the excessive intake of fat, especially saturated fat, caused by the intake of animal protein in humans. More importantly, soy protein can keep the blood glucose level and blood lipids of diabetic patients normal, reduce oxidative stress, inhibit attacks from AGEs, and prevent diabetic patients from developing complications. A low-protein diet is meaningful in bringing glomerular filtration closer to normal and reducing the symptoms of uremia. Research on chronic kidney disease has shown that a low-protein diet may also cause malnutrition, which is a risk factor for death. Therefore, a low-protein diet has both beneficial and non-beneficial effects on the kidney.

Results from animal studies have shown that isoflavones inhibit the progression of diabetic nephropathy. In STZ-induced diabetic models, alloxan-induced diabetic mice, and experimental models of nephrotic syndrome, administration of genistein reduces renal fibrosis and inflammation. The mechanism has been shown to be through the suppression of oxidative stress via activation of Nrf2 and the inhibition of TGF-β and NF-κB. Similarly, administration of daidzein in a streptozotocin-induced diabetic model led to improved renal histology. It also led to a significant decrease in creatinine and blood urea nitrogen levels, and an increase in the expression levels of antioxidant enzymes such as glutathione, catalase, and superoxide dismutase [99]. Formononetin treatment for 16 weeks led to improved glycemic parameters and a significant increase in creatinine clearance in the same experimental model, and it was associated with increased *sirtuin* (*SIRT*)*1* expression in the kidney [105]. *SIRT1* is a gene involved in the development of diabetic nephropathy, in which persistent hyperglycemic conditions damage the kidney’s small blood vessels, causing glomeruli to break down and waste products to leak into the urine. If this condition continues for a long time, kidney function may decline and progress to renal failure, requiring hemodialysis. Furthermore, isoflavone supplementation reduced urinary albumin excretion, reduced the urinary albumin-to-creatinine ratio, and delayed the progression of diabetic nephropathy in a diabetic db/db mouse model.

## 7. Conclusions and Future Directions

Diabetic nephropathy often leads to end-stage renal disease [106].

We have previously conducted cell and animal experiments using molecular biology techniques, staining techniques, and liquid chromatography mass spectrometry (LC/MS) to reveal the basic mechanisms underlying diabetic complications—particularly diabetic nephropathy—in order to investigate the significance of the antioxidant properties of food components such as polyphenols in this context.

We reported the importance of urinary angiotensinogen as an early marker for the onset of diabetic nephropathy and showed that it increases more sensitively than urinary albumin. We also suggested that angiotensinogen in the kidney is linked to oxidative stress, in the process of verifying whether the amount of AGT excreted in urine changes in correlation with oxidative stress. Furthermore, through clarifying how the intake of soy isoflavones—which possess antioxidant properties—affects the progression of diabetic nephropathy via AGT in detail, the usefulness of soy isoflavones in slowing or reversing the progression of diabetic nephropathy can be elucidated. In the future, progress is expected in research on the clinical verification of urinary AGT, synergistic effects of isoflavones with other polyphenols, and AGT-targeted therapy. Our findings are expected to contribute to the development of dietary therapies to address the progression mechanism of diabetic nephropathy, which is an international issue. It is also important that large-scale studies on such dietary therapies—including clinical research—be organized in various countries, which are expected to produce results that confirm the mechanisms through which polyphenols inhibit the progression of diabetic nephropathy, as discussed in this study.

## Figures and Tables

**Table 1:** Biomarkers for diabetic nephropathy.

Biomarkers	Markers in Urine, Serum, or Urine/Serum	
Glomerular injury markers		
Albumin Moresco, R. N. et al. [50]	Urine	Urinary albumin levels within the microalbuminuria stage are predictive of end-stage renal disease. There is a large variability and low specificity for diabetic nephropathy.
Oxidized albumin Watanabe, H. et al. [51]	Serum	Oxidized albumin levels may be useful for the early diagnosis of diabetic kidney disease and predicting renal outcomes.
Type IV collagen Iijima, T. et al. [52]	Urine	Biomarker for the early stages of diabetic nephropathy.
Ceruloplasmin Lee, M. J. et al. [53], Hellemons, M. E. et al. [54]	Serum	Serum ceruloplasmin is an independent predictor of the progression of diabetic nephropathy in patients with type 2 diabetes.
Markers of renal tubular injury		
Angiotensinogen Kamiyama et al. [14], Sun, W. et al. [15], Wang, J. et al. [16] Zhuang, Z. et al. [55]	Urine	Biomarker for the early phases of diabetic nephropathy.
α_1_-MG Zhou, Y. et al. [57]	Urine	Urinary α_1_-MG (which measures proximal tubular dysfunction) is useful for the early detection of nephropathy in diabetic subjects.
KIM-1 Fiseha, T. et al. [58]	Serum and urine	KIM-1 is a sensitive and specific marker of kidney injury, as well as a predictor of prognosis.
L-FABP Liu, H. et al. [59]	Urine	L-FABP is an independent predictor of the progression of DN irrespective of disease stage.
NGAL Motawi et al. [60], Veiga et al. [61]	Serum and urine	NGAL can predict albuminuria and be used as a non-invasive tool for the diagnosis, staging, and progression of diabetic nephropathy.
NAG Fiseha, T. et al. [58]	Urine	Urinary NAG reflects the degree of renal impairment in diabetic nephropathy.
Inflammatory markers		
Inflammatory cytokines		
IL-6 Ahmed, S. A. et al. [62]	Serum and urine	Signaling of inflammatory cytokines participates in inflammation responses central to the progression of diabetic nephropathy.
IL-8 Karimi, F. et al. [56]	Serum and urine	
IL-10 Karimi, F. et al. [56]	Serum and urine	
IL-18 Ahmed, S. A. et al. [62]	Serum and urine	
TNF-α Ahmed, S. A. et al. [62]	Serum and urine	
Growth factors		
TGF-β Wang, L. et al. [63]	Urine	TGF-β is a pleiotropic cytokine, which has been recognized as a key mediator of diabetic nephropathy.
CTGF Gilbert, R. E. et al. [64]	Serum and urine	CTGF is a biomarker reflecting both glomerular and tubulointerstitial hallmarks of diabetic kidney disease.
Adhesion molecules		
ICAM-1 Duran-Salgado, M. B. et al. [65]	Serum	ICAM1 is a potential biomarker and target for the prediction and treatment of diabetes and diabetic nephropathy.
VCAM-1 Deng, Y. et al. [66]	Serum	VCAM-1 indicates microvascular complication among patients with type 2 diabetes.
Fetuin-A Inoue, K. et al. [67]	Serum and urine	Fetuin-A is a risk factor for diabetic nephropathy with microalbuminuria or GFR < 60 mL/min.
Oxidative stress		
8-hydroxy-2′-deoxyguanosine (8-OHdG) Wu, L. L. et al. [68]	Serum	Serum 8-OHdG is a potential biomarker for assessing oxidative stress and DNA damage in patients with diabetes and renal complications.
Pentosidine Kerkeni, M. et al. [69]	Serum and urine	Pentosidine levels may be a biomarker for microvascular complications in type 2 diabetic patients.
CKD markers		
Cystatin C Khosravi, N. et al. [70] Benoit, S. W. et al. [71]	Serum and urine	Serum cystatin C is a useful marker of early renal impairment in type 2 diabetic patients, as it reflects both a decrease in GFR and an elevated albumin-to-creatinine ratio.

**Table 2:** The roles of isoflavones in the progression of diabetic nephropathy.

Authors	Animals/Isoflavone	Function
Jheng H.F. et al. [94]	KKAy mice/genistein	Regression of fibrosis
Yang S. et al. [95]	db/db mice/tectorigenin	Regression of fibrosis
Amin F.M. et al. [96]	db/db mice/piperine	Regression of mitigation of aortic vasculopathy
Li Y. et al. [97]	Sprague Dawley (SD) rats with diabetic nephropathy/genistein	Regression of mitigation of aortic vasculopathy mitochondrial function and inflammation
Kim, M. J. et al. [98]	Alloxan-injected mice/genistein	Regulation of oxidative stress and inflammation
Jia, Q. et al. [99]	Streptozotocin (STZ) rats/genistein	Alleviation of renal fibrosis

## Abbreviations

PKC: protein kinase C; RAS: renin–angiotensin system; AGT: angiotensinogen; ACE: angiotensin-converting enzyme; IVD: intervertebral disc; SGLT2: sodium–glucose cotransporter-2; TGF-β: transforming growth factor-beta; SIRT1: sirtuin 1; MAP: mitogen-activated protein; NF-κB: nuclear transcription factors; VEGF: vascular endothelial growth factor; α1-MG: α1-microglobulin; KIM-1: kidney injury molecule-1; L-FABP: L-fatty acid binding protein; NGAL: neutrophil gelatinase-associated lipocalin; NAG: N-acetyl-beta-D-glucosaminidase; H-FABP: heart-type fatty acid-binding protein; IL: interleukin; CTGF: connective tissue growth factor; VCAM-1vascular cell adhesion molecule-1; ICAM-1: intercellular adhesion molecule-1; GFR: glomerular filtration rate; AGEs: advanced glycation end products; CML: carboxymethyllysine; CEL: carboxyethyllysine; 8-OHdG: 8-hydroxy-2′-deoxyguanosine; Smad2: SMAD family member 2; SD: Sprague Dawley; STZ: streptozotocin; BUN: blood urea nitrogen; FBG: fibrinogen; LDL-C: low-density lipoprotein cholesterol; Scr: serum creatinine; CrCl: creatinine clearance; GFR: glomerular filtration rate; HDL-C: high-density lipoprotein cholesterol; LC/MS: liquid chromatography mass spectrometry.
